# Comparing Different Anesthesia Methods on Anesthetic Effect and Postoperative Pain in Patients with Early Gastric Cancer during Endoscopic Submucosal Dissection

**DOI:** 10.1155/2022/7299360

**Published:** 2022-08-29

**Authors:** Jie Zhang, Yanlei Chen, Zhiwu Liu, Zhihao Pan

**Affiliations:** ^1^Department of Anesthesia, Yantai Mountain Hospital, Yantai 264001, Shangdong, China; ^2^Chinese Peoples Liberat Army, Dept of Gastroenterology, Hosp970, Joint Logist Support Unit, Yantai 264001, Shangdong, China; ^3^Chinese Peoples Liberat Army, Dept Anesthesiol, Hosp 970, Joint Logist Support Unit, Yantai 264001, Shangdong, China

## Abstract

Endoscopic submucosal dissection (ESD) is a minimally invasive technique to completely peel the pathological mucosa from the submucosa under endoscopy, which has been often utilized to treat early gastric cancer. During the operation, anesthesia is required to reduce the discomfort due to the complexity, high risk, and longtime operation of ESD. In this study, we compared different anesthesia methods on anesthetic effect and postoperative pain in patients (≥65 years old) with early gastric cancer during ESD. For this purpose, 60 patients with early gastric cancer who were more than 65 years old were selected from January 2019 to December 2021, where 30 patients treated with simple intravenous general anesthesia were divided into the simple group and 30 patients treated with intravenous combined inhalation general anesthesia were regarded as the composite group. The hemodynamic index, wake-up time, postoperative pain intensity, operation time, and the incidence of adverse reactions were compared between the two groups. For the hemodynamic index before incision, after incision, and at the end of the operation, the mean arterial pressure (MAP) in the composite group was higher than that in the simple group (*P* < 0.05) and the heart rate (HR) was lower than that in the simple group (*P* < 0.05). After the ESD operation, the wake-up time and visual analogue scale (VAS) in the composite group were lower than those in the simple group (*P* < 0.05). In addition, the ESD operation time and incidence of adverse reactions in the composite group was significantly lower than that in the simple group (*P* < 0.05). These results showed that intravenous combined inhalation general anesthesia had a good anesthetic effect, stable hemodynamics during ESD operation, and slight postoperative pain.

## 1. Introduction

Early gastric cancer refers to the early stage of gastric cancer, where the lesion only involves the mucosa or submucosa and does not consider the size of the lesion and lymph node metastasis [1]. It is difficult to detect since it has no obvious symptoms or sometimes has nonspecific symptoms such as epigastric discomfort, satiety after eating, nausea, and so on [2]. More than 75% of patients have entered the advanced stage when they are first diagnosed with gastric cancer and the mortality of late gastric cancer is high [3]. With the development of medical technology, endoscopic submucosal dissection (ESD) have been used to diagnose and treat early gastric cancer, which increased the survival rate and survival time. ESD indicated the minimally invasive technique to completely peel the pathological mucosa from the submucosa under endoscopy [4–6]. Many studies have shown that ESD is not inferior to surgery in terms of complete resection rate, and it has the advantages of minimally invasive, short hospital stay, and low total treatment cost [7, 8].

The ESD operation is carried out under anesthesia, since the patients are very likely to have bleeding, perforation, and other phenomena, and even circulatory and respiratory dysfunction during a long time of the ESD operation [9, 10]. Common anesthesia methods include simple intravenous general anesthesia (propofol plus small dose fentanyl) and intravenous combined inhalation general anesthesia [11, 12]. Propofol plus low-dose fentanyl sedation is often used in short-term outpatient surgery, such as gastrointestinal endoscopy and induced abortion, which has the characteristics of rapid onset, high quality of recovery, and low postoperative adverse reactions [13]. Intravenous combined inhalation general anesthesia refers to the method of combining intravenous anesthetics with inhalation anesthetics to produce and maintain general anesthesia. Intravenous anesthetics are often used to induce anesthesia because of their fast onset and no irritation to respiratory tract, and inhaled anesthetics are usually used to maintain general anesthesia since it is easy to control the anesthesia and recover after operation [14, 15]. For the patients more than 65 years old, surgical anesthesia is a big problem. Specifically, elderly patients often suffer from one or several chronic diseases due to functional decline of various organs and low immunity, resulting in poor tolerance to surgery and anesthesia, strong stress response, unstable intraoperative hemodynamics, and increased anesthesia risk [16, 17]. Therefore, it is very important and necessary to explore an effective anesthesia method.

Here, we chose 60 elderly patients with early gastric cancer and compared the effects of two different anesthesia methods to clarify the application advantages of intravenous combined inhalation general anesthesia and its impact on the anesthetic effect and postoperative pain of patients.

## 2. Materials and Methods

### 2.1. Clinical Data

**Figure 1:**
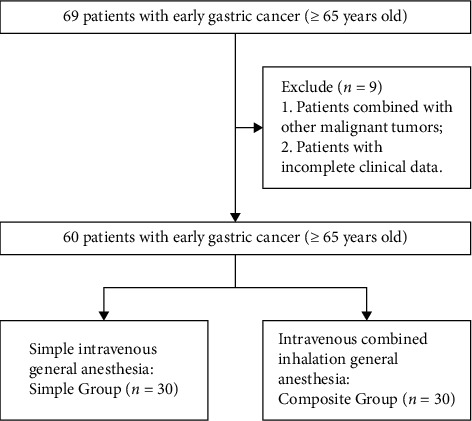
The flowchart of patients' selection and classification.

69 patients hospitalized in our hospital from January 2019 to December 2021 with early gastric cancer who were more than 65 years old were selected retrospectively. After filtering by the inclusion and exclusion criteria, 60 patients were kept to be further analyzed, where 30 patients treated with simple intravenous general anesthesia (propofol plus small dose fentanyl) were divided into the simple group and other 30 patients treated with intravenous combined inhalation general anesthesia were divided to the composite group (Figure 1). The study protocol has been approved by the ethics committee of our hospital.

#### 2.1.1. Inclusion Criteria

The inclusion criteria were as follows:

1. All the patients fit the diagnostic criteria for early gastric cancer: the tumor tissue was limited to the gastric mucosa or submucosa, the diameter of small gastric cancer lesions was ≤5 mm, the diameter of small gastric cancer lesions was 5∼10 mm, and the diagnosis was made by endoscopy and pathological biopsy.
2. Age ≥65 years old.
3. Patients complying with ESD treatment indications.
4. Patients carried on the ESD treatment successfully.
5. Patients with complete clinical medical records.

#### 2.1.2. Exclusion Criteria

The exclusion criteria were as follows:

1. Patients combined with other malignant tumors
2. Patients with incomplete clinical medical records

### 2.2. ESD Treatment

All patients were treated with ESD. Food and water were forbidden for 6 h and 2 h at least, respectively, before operation. After general anesthesia, the lesions were located under routine endoscopy. In combination with iodine staining and magnifying endoscopy, the lesion boundary was confirmed. Spot electrocoagulation marks were made at the place 3∼5 mm away from the lesion edge. The submucosal injection was made at the outside of the mark point with 10% glycerol fructose concrete solution, and the lesion boundary was fully raised. We cut the lesion mucosa to the submucosa circularly at about 5 mm outside the marked point and gradually peel off along the submucosa below the lesion until the lesion mucosa completely falls off. After the lesion mucosa is completely stripped, argon ion coagulation should be performed to treat the visible small blood vessels on the wound surface. For those with deep local stripping, intrinsic muscular injury, and visible holes, metal clips should be used to clamp them.

### 2.3. Anesthesia

#### 2.3.1. The Anesthesia in the Simple Group

30 patients in the simple group were treated with simple intravenous general anesthesia (propofol plus small dose fentanyl). Specifically, continuous oxygen inhalation through nasal catheter, oxygen flow rate of 8l/min, intravenous injection of fentanyl 1µg/kg, and propofol 2∼2.5 mg/kg for induction. The operation was started after the patient fell asleep and the eyelash reflex disappeared. During the operation, propofol 4∼6 mg/(kg h) was continuously pumped to maintain anesthesia until the end of the operation.

#### 2.3.2. The Anesthesia in the Composite Group

30 patients in the composite group were treated with intravenous combined inhalation general anesthesia. Anesthesia induction: intravenous injection of 1∼2 mg/kg propofol, 1∼1.5 mg/kg vecuronium, and 4∼6 µg/kg fentanyl. Anesthesia maintenance: after endotracheal intubation, propofol (10 g/L) was injected intravenously at the rate of 15∼20 ml/h with a micropump, sevoflurane (volume fraction: 0.015∼0.020) was inhaled, and fentanyl was intermittently injected intravenously.

### 2.4. Observation Index

The observation indexes were as follows:

1. The general data including age and gender
2. Hemodynamic index including the mean arterial pressure (MAP), heart rate (HR) at 30 min and 60 min, and the end of the operation
3. The wake-up time
4. Postoperative pain: visual analogue scale (VAS) [18] was utilized to measure the pain at 1, 6, 12, and 24 hours after the operation. The full score was 10, and the lower the score, the less the pain

### 2.5. Statistical Processing

SPSS 26.0 and GraphPad Prism 8.0 were utilized to analyze and visualized the data, respectively. The measurement data were expressed in (mean ± sd) and analyzed by *T* test, which was consistent with the normal distribution. The counting data were expressed in the number of cases and compared by *χ*^2^ test. *P* < 0.05 reveals that there is an obvious difference between the groups.

## 3. Results

### 3.1. Clinical Data in the Simple and Composite Groups

As shown in Table 1, for the age, gender, BMI, and ASA, there was no obvious distinction between the simple and composite groups (*P* > 0.05), while the ESD operation time in the composite group was shorter than that in the simple group (*P* < 0.05).

### 3.2. Comparison of MAP

**Figure 2:**
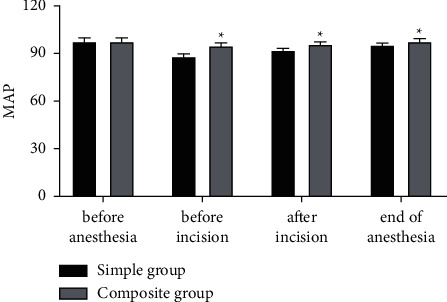
Comparison of MAP (mmHg). ^*∗*^represents *P* < 0.05 compared with the simple group.

Before anesthesia, the MAP in the simple and composite groups was no difference (*P* > 0.05). Before incision, after incision, and at the end of anesthesia operation, the MAP in the composite group was higher than that in the simple group (*P* < 0.05) (Figure 2). Besides, the fluctuation amplitude of MAP in the composite group was smaller than that in the simple group, indicating that the MAP in the group with intravenous combined inhalation general anesthesia was more stable.

### 3.3. Comparison of HR

**Figure 3:**
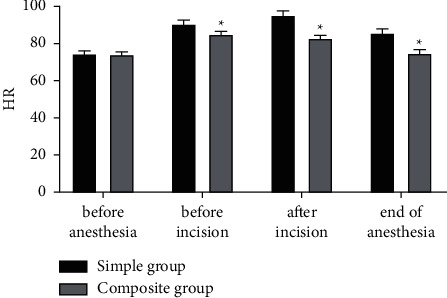
Comparison of HR (mmHg). ^*∗*^represents *P* < 0.05 compared with the simple group.

There was no difference in the HR between the two cohorts before anesthesia (*P* > 0.05). Before incision, after incision, and at the end of anesthesia the HR in the composite group was obviously lower than that in the simple group (*P* < 0.05) (Figure 3). Additionally, the fluctuation amplitude of HR in the composite group was smaller than that in the simple group, indicating that the HR in the group with intravenous combined inhalation general anesthesia was more steady.

### 3.4. Comparison of the Wake-Up Time

**Figure 4:**
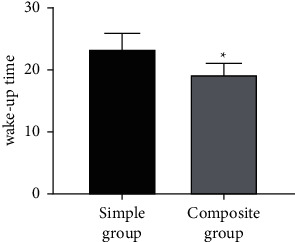
Comparison of the wake-up time (min). ^*∗*^represents *P* < 0.05 compared with the simple group.

Figure 4 reveals that the wake-up time in the simple and composite groups was 23.23 ± 2.648 (min) and 19.27 ± 1.760 (min), respectively, indicating that the wake-up time in the composite group was shorter than that in the simple group (*P* < 0.05).

### 3.5. Comparison of the VAS Score

**Figure 5:**
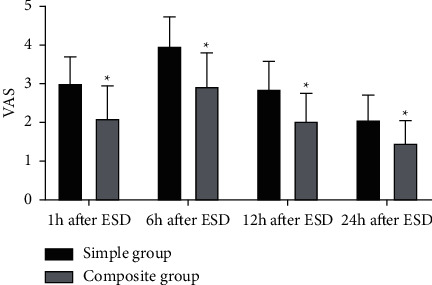
Comparison of VAS. ^*∗*^represents *P* < 0.05 compared with the simple group.

As shown in Figure 5, at 1, 6, 12, and 24 hours after the ESD, the VAS scores in the composite group were lower than those in the simple group (*P* < 0.05), indicating that the analgesic effect of intravenous combined inhalation general anesthesia was better than simple intravenous general anesthesia.

### 3.6. Comparison of the Adverse Reactions

We further collected the data of patients' adverse reactions. Specifically, there were 4 cases of body movement, 3 cases of hypoxemia, and 2 cases of cough in the simple group. A total of 2 patients had adverse reactions in the composite group, one was cough, and the other was hypoxemia. The comparison result shows that the incidence of adverse reactions in the composite group was significantly lower than that in the simple group (*P* < 0.05) as shown in Table 2.

## 4. Discussion

At present, gastric cancer is still endangering human health, with a high degree of malignancy and poor prognosis [19]. Among malignant tumors, its incidence rate and mortality ranked second and third in China [20, 21]. With the change of population aging, elderly patients have become the main population of gastric cancer, accounting for about two-thirds of the total number of patients [22, 23]. In recent years, with the popularization of gastroscopic examination and the increase of people's attention to their own health, more and more gastric cancer lesions can be diagnosed by endoscopy in the early stage [24, 25]. Compared with traditional radical surgical resection, early gastric cancer located in the mucosa or submucosa can be treated with minimally invasive endoscopic submucosal dissection [26]. During the operation, scientific and effective anesthesia is the key to ensuring the smooth implementation of radical gastrectomy for gastric cancer and achieving ideal results [27]. Therefore, the choice of safe and efficient anesthesia has an important clinical significance and value.

The commonly used anesthesia methods are propofol plus low-dose fentanyl intravenous analgesia and intravenous combined inhalation anesthesia [28]. Propofol plus low-dose fentanyl sedation is commonly used in short-term outpatient surgery, such as gastrointestinal endoscopy and induced abortion. It has the characteristics of rapid onset, high quality of recovery, and small postoperative adverse reactions [29–31]. Our results showed that the MAP decreased and HR increased during operation compared with those before ESD, which may be related to the decreased sympathetic tension and vasodilation of propofol after the patient falls asleep [32].

In addition, the results of this study revealed that the fluctuation amplitude of MAP and HR in the composite group was less than that in the simple group, suggesting that intravenous combined inhalation can maintain the stability of hemodynamics during operation compared with simple intravenous general anesthesia. Intravenous combined inhalation general anesthesia can effectively protect the airway and carry out mechanical ventilation, so it can maintain the appropriate depth of anesthesia, so as to avoid adverse reactions in simple intravenous general anesthesia, improve oxygenation, and maintain hemodynamic stability [33]. Propofol, sevoflurane, and fentanyl were used in this study, which have the characteristics of fast onset, short action time, and no accumulation and meet the requirements of “fast channel anesthesia”. [34]. This study also showed that the wake-up time of the composite group is shorter than that of the simple group, and the postoperative pain intensity is significantly lower than that in the simple group. Our result showed that the incidence of adverse reactions in the composite group was significantly lower than that in the simple group, indicating that intravenous combined inhalation general anesthesia can decrease the wake-up time of patients and create good conditions for postoperative recovery.

In conclusion, compared with simple intravenous general anesthesia, intravenous combined inhalation general anesthesia can better maintain the intraoperative hemodynamic stability of patients with early gastric cancer, shorten the postoperative recovery time, and reduce the postoperative pain. On the other hand, this study is a retrospective study, which may have some limitations. The analysis of the indicators is less and there are many variables between the comparison of two groups, leading to the comparison of the two anesthesia methods is not comprehensive enough. In addition, the number of objects in our study is small, and there may be a sample deviation. In the next study, prospective research is needed to obtain more comprehensive evaluations and more credible conclusions.

## Figures and Tables

**Table 1:** Clinical data in the simple and composite groups.

Index	Simple group (*n* = 30)	Composite group (*n* = 30)	*t*/*χ*^2^	*P*
Age (years)	75.07 ± 4.127	75.17 ± 4.194	−0.093	0.926
Sex (male, %)	16 (53.33%)	15 (50%)	0.067	0.796
BMI (kg) (m^2^)	20.47 ± 0.603	20.45 ± 0.625	0.137	0.892
ASA (I, %)	15 (50.00%)	16 (53.33%)	0.067	0.796
ESD time (h)	2.31 ± 0.180	1.78 ± 0.158	12.200	*P* < 0.001

**Table 2:** Comparison of the adverse reactions.

Group	Body movement	Hypoxemia	Cough	Total
Simple group	4	3	2	9
Composite group	1	1		2
*χ* ^2^				5.455
*P*				0.020

## Data Availability

The raw data used to support the findings of this study could be obtained by getting in touch the corresponding author.
